# Patient With Tirzepatide-treated Type 2 Diabetes With Difficult Visualization During Esophagogastroduodenoscopy

**DOI:** 10.1210/jcemcr/luaf044

**Published:** 2025-03-21

**Authors:** Hiroki Dobashi, Daiki Shioya, Koji Kikkawa, Kihachi Ohshima, Junichi Okada, Shuichi Okada

**Affiliations:** Diabetes Center, Hidaka Hospital, Takasaki, Gunma 370-0001, Japan; Diabetes Center, Hidaka Hospital, Takasaki, Gunma 370-0001, Japan; Diabetes Center, Hidaka Hospital, Takasaki, Gunma 370-0001, Japan; Diabetes Center, Hidaka Hospital, Takasaki, Gunma 370-0001, Japan; Department of Medicine, Division of Endocrinology, Albert Einstein College of Medicine, Bronx, NY 10461, USA; Diabetes Center, Hidaka Hospital, Takasaki, Gunma 370-0001, Japan

**Keywords:** tirzepatide, glycated hemoglobin, type 2 diabetes mellitus, esophagogastroduodenoscopy

## Abstract

Tirzepatide, a dual glucagon-like peptide-1 receptor and gastric inhibitory peptide receptor agonist, is an effective treatment for type 2 diabetes mellitus and obesity, resulting in significant improvements in glycated hemoglobin levels and weight reduction. However, gastrointestinal side effects, including delayed gastric emptying, have been reported. Recently, a case of inadequate visualization during esophagogastroduodenoscopy due to substantial food residues in patients treated with tirzepatide has been reported. This study aims to report a patient who underwent a successful esophagogastroduodenoscopy following a 2-month discontinuation of tirzepatide. Previously, the same patient experienced incomplete esophagogastroduodenoscopies due to excessive food residues despite standard preparation. The findings of this study emphasize the challenges associated with the use of tirzepatide for upper gastrointestinal procedures and suggest that the discontinuation of the drug might be necessary to ensure optimal endoscopic evaluation.

## Introduction

Glucagon-like peptide-1 receptor agonists (GLP-1 RAs) are commonly used to treat type 2 diabetes mellitus (T2D), but their effects on gastric mortality can lead to residual gastric contents that interfere with esophagogastroduodenoscopies [1, 2]. Tirzepatide, a dual glucagon-like peptide-1 and gastric inhibitory peptide receptor agonist, is also widely used to treat T2D, obesity, and sleep apnea syndrome caused by severe obesity. However, tirzepatide has also been reported to have several side effects including nausea, vomiting, anorexia, and delayed gastric emptying. This report aims to discuss a unique case in which tirzepatide therapy significantly impacted the outcomes of an esophagogastroduodenoscopy, providing insights into managing such cases.

## Case Presentation

A 65-year-old male was diagnosed with T2D at age 42 (body height, 173.8 cm; body weight, 79.8 kg; body mass index, 26.4 kg/m^2^); he underwent a kidney transplant for diabetic nephropathy at age 50. His medical history included mild diabetic neuropathy, proliferative diabetic retinopathy (treated with laser therapy), diabetic nephropathy of G3bA2, and reflux esophagitis. His mother was being treated for T2D.

Table 1 summarizes the patient's casual peripheral blood laboratory findings before the initiation of tirzepatide. The main findings were as follows: aspartate aminotransferase 17 IU/L (normal range, 10-40 IU/L), alanine aminotransferase 18 IU/L (normal range, 5-45 IU/L), γ-glutamyl transpeptidase 19 IU/L (normal range, 0-79 IU/L), triglyceride 1.67 mmoL/L (148 mg/dL) (normal range, < 1.98 mmoL/L [<175 mg/dL]), high-density lipoprotein 1.70 mmoL/L (66 mg/dL) (normal range, 1.0-2.1 mmoL/L [40-80 mg/dL]), low-density lipoprotein 2.87 mmoL/L (111 mg/dL) (normal range, 1.81-3.59 mmoL/L [70-139 mg/dL]), blood urea nitrogen 8.5 mmoL/L (23.8 mg/dL) (normal range, 2.86-7.14 mmoL/L [8.0-20.0 mg/dL]), serum creatinine 112.2 mmol/L (1.27 mg/dL) (normal range, 57.5-96.4 mmoL/L [0.65-1.09 mg/dL]), estimated glomerular filtration rate 45.0 mL/min/1.73m^2^, urinary albumin/creatinine ratio 121.3 mg/gCr (normal range, <30 mg/gCr), casual plasma glucose 5.88 mmoL/L (106 mg/dL), and casual serum insulin 8.2 μU/mL. His urine was 4+ for glucose (normal range, –), positive for protein (normal range, –∼±), and negative for ketone body (normal range, –), while the red (normal range, 0-4 HPF) and white blood cell (normal range, 0-4 HPF) were not identified in the urine sediment. His chest X-ray examination and electrocardiogram results were normal. Moreover, his anti-glutamic acid decarboxylase antibody level was <5.0 U/mL (normal range, <5.0 U/mL).

**Table 1.** Patient's peripheral blood laboratory findings

Clinical test items	Measured value	Normal range
AST	17 IU/L	10-40 IU/L
ALT	18 IU/L	5-45 IU/L
γ-GTP	19 IU/L	0-79 IU/L
TG	148 (1.67) mg/dL (<1.98 mmoL/L)	<175 mg/dL (<1.98 mmoL/L)
HDL	66 (1.70) mg/dL (1.0-2.1 mmoL/L)	40-80 mg/dL (1.0-2.1 mmoL/L)
LDL	111 (2.87) mg/dL (1.81-3.59 mmoL/L)	70-139 mg/dL (1.81-3.59 mmoL/L)
BUN	23.8 (8.5) mg/dL (2.86-7.14 mmol/L)	8-20 mg/dL (2.86-7.14 mmol/L)
Cre	1.27 (112.2) mg/dL (57.7-96.4 μmol/L)	0.65-1.09 mg/dL (57.7-96.4 μmol/L)
eGFR	45 mL/min/1.73m^2^	>60 (mL/min/1.73m^2^)
UA	6.7 (398.5) mg/dL (214.1-416.4 μmoL/L)	3.6-7.0 mg/dL (214.1-416.4 μmoL/L)
Na	141 (141) mEq/L (135-145 mmoL/L)	135-145 mEq/L (135-145 mmoL/L)
K	4.1 (4.1) mEq/L (3.5-5.0 mmoL/L)	3.5-5.0 mEq/L (3.5-5.0 mmoL/L)
CL	104 (104) mEq/L (98-108 mmoL/L)	98-108 mEq/L (98-108 mmoL/L)
Serum amylase	77 IU/L	39-134 IU/L
WBC	6860 /mL	3500-9700 /mL
RBC	478 × 10^4^/mL	438-577 × 10^4^/mL
Hb	13.5 (135) g/dL (136-183 g/L)	13.6-18.3 g/dL (136-183 g/L)
Ht	43.5 (0.435) % (0.405-0.519/L)	40.5-51.9 % (0.405-0.519/L)
Platelet	14.3 × 10^4^/mL	14.0-37.9 × 10^4^/mL
GAD antibody	<0.5 U/mL	<0.5 U/mL
Casual plasma glucose	106 mg/dL	mg/dL
Casual serum insulin	8.2 μU/mL	μU/mL
Urinary albumin/creatinine ratio	121.3 mg/gCr	<30 mg/gCr
Clinical test items (urinalysis)		
Protein	±	– ∼ ±
Occult blood	–	–
Ketone body	–	–
Urinary sediment		
RBC	0-1/HPF	1-4/HPF
WBC	1-4/HPF	1-4/HPF

Values in parentheses are International System of Units.

Abbreviations: ALT, alanine aminotransferase; AST, aspartate aminotransferase; BUN, blood urea nitrogen; CL, chloride; Cre, serum creatinine; eGFR, estimated glomerular filtration rate; GAD, anti-glutamic acid decarboxylase; Hb, hemoglobin; HDL, high-density lipoprotein; HPF, high power field; Ht, hematocrit; K, potassium; LDL, low-density lipoprotein; Na, sodium; RBC, red blood cell count; TG, triglyceride; UA, uric acid; WBC, white blood cell count; γ-GTP, γ-glutamyl transpeptidase.

## Diagnostic Assessment

He had no history of diabetic ketoacidosis and had never been treated with insulin until now. During the screening examination at our hospital, his anti-glutamic acid decarboxylase antibody level was <5.0 U/mL, while his diabetes was classified as T2D.

## Treatment

He was treated with dapagliflozin (10 mg/day), mitiglinide (60 mg/day), and linagliptin (5 mg/day). Subsequently, 2.5 mg of tirzepatide was used in place of linagliptin. One month later, 2.5 mg of tirzepatide was switched to 5.0 mg of tirzepatide. Every time the patient visited our hospital, a qualified dietician, registered nurse and physician gave him advice and recommendations on his diet and lifestyle modifications. Furthermore, no drugs were prescribed to treat the digestive system, including drugs that affect the motor function of the gastrointestinal tract.

## Outcome and Follow-up

In March 2024, the patient’s glycated hemoglobin (HbA1c) level was 96.2 mmoL/moL (8.8%) before the initiation of tirzepatide. In September 2024 (6 months after the initiation of tirzepatide), his HbA1c level was 75.5 mmoL/moL (6.9%). Unfortunately, his HbA1c level increased to 93.4 mmoL/moL (8.5%) after 2 months of discontinuing tirzepatide for esophagogastroduodenoscopy. However, 1 month after resuming tirzepatide, his HbA1c level had decreased to 80.3 mmoL/moL (7.3%).

The patient reported no gastrointestinal symptoms or other side effects while using tirzepatide, as was the case before he used tirzepatide. He was also regularly administered tirzepatide.

This patient had undergone annual physical examinations at our hospital, including follow-up of reflux esophagitis via esophagogastroduodenoscopy.

**Figure 1.**
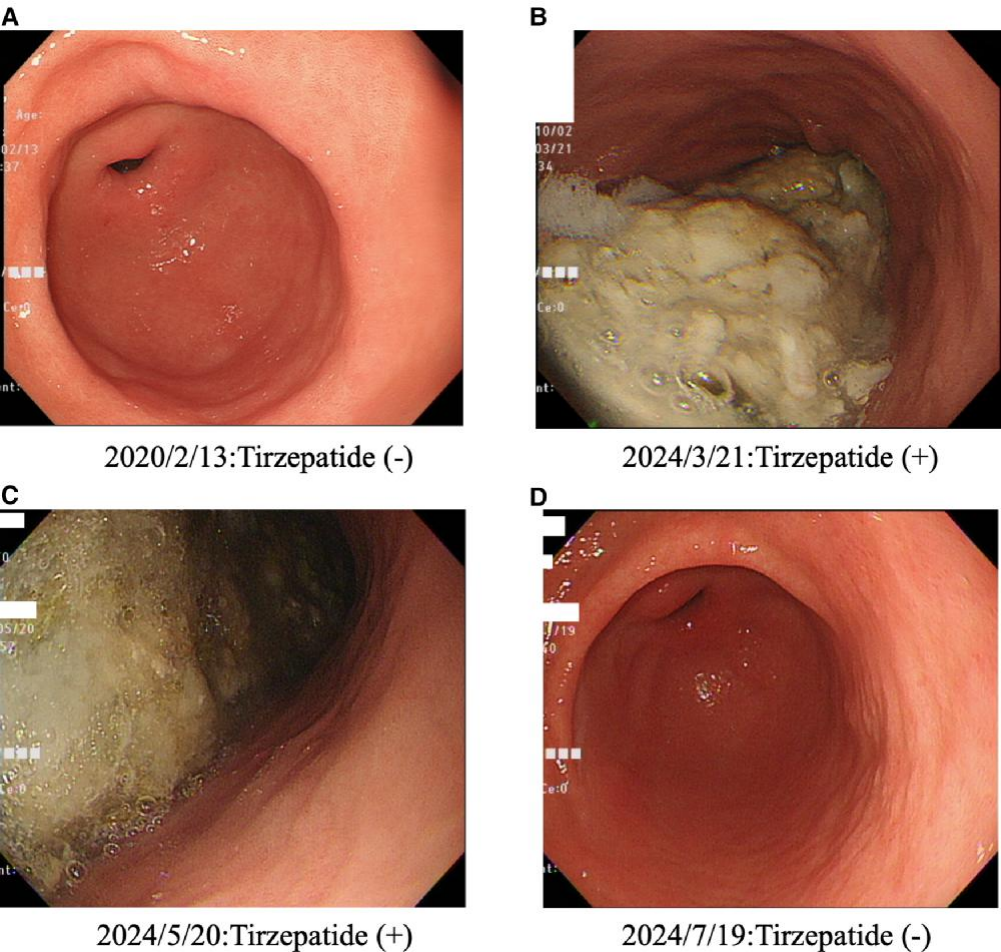
Esophagogastroduodenoscopic findings with or without tirzepatide. (A) In 2020, the esophagogastroduodenoscopic findings included grade A reflux esophagitis; however, no other abnormal findings were indicated. (B-C) In March and May 2024, the esophagogastroduodenoscopic findings were incomplete due to the presence of large amounts of food residues associated with the use of tirzepatide. Other esophagogastroduodenoscopic findings were similar to those observed in 2020. (D) The esophagogastroduodenoscopic findings were back to normal after 2 months of the discontinuation of tirzepatide (July 2024). Other esophagogastroduodenoscopic findings were similar to those observed in 2020.

The following were the results of the esophagogastroduodenoscopy. In 2020, the esophagogastroduodenoscopic findings revealed no signs of tirzepatide (Fig. 1A). Other esophagogastroduodenoscopic findings included grade A reflux esophagitis; however, no other abnormal findings were observed. However, due to the high levels of food residues associated with the use of tirzepatide in March 2024, the results of the esophagogastroduodenoscopies were incomplete (Fig. 1B). Other esophagogastroduodenoscopic findings were similar to those in 2020. The patient was very intent on confirming the exact esophagogastroduodenoscopic findings in the areas that could not be observed. Thus, we decided to reexamine the patient via esophagogastroduodenoscopy. However, due to the high levels of food residues associated with the use of tirzepatide in May 2024, the results of the esophagogastroduodenoscopies were again incomplete (Fig. 1C). The results revealed that with the use of tirzepatide, the normal pretreatment was not sufficient for esophagogastroduodenoscopy in this patient. At this point, it has not been determined how long the patient needs to be off tirzepatide to be able to perform the esophagogastroduodenoscopy without problems, so to be on the safe side, we consulted with the patient and decided to take the patient off tirzepatide for 2 months. Of note, 2 months after discontinuing tirzepatide (July 2024), the esophagogastroduodenoscopic findings were back to normal (Fig. 1D). Other esophagogastroduodenoscopic findings were similar to those in 2020. Fortunately, we did not observe aspiration pneumonia in this case.

## Discussion

The dual incretin receptor agonist tirzepatide and GLP-1 RAs have a significant and growing influence on the current management of T2D and obesity. In addition to their strong glucose-lowering properties, GLP-1 RAs help obese people lose weight, reduce their risk of cardiovascular and renal illness, and possibly alleviate steatotic liver disease linked to metabolic dysfunction [3]. GLP-1RAs tend to affect gastric, intestinal, and gallbladder motility. This can be advantageous, for example, by delaying the entry of glucose and lipids into the bloodstream after meals, but this can also increase the risk of negative outcomes, such as retained gastric contents, which could lead to pulmonary aspiration during endoscopy or general anesthesia induction, as well as gallbladder and biliary complications.

The impact of diabetes mellitus itself (ie, in the absence of GLP-1 RA treatment) on retained gastric contents and pulmonary aspiration has not been further investigated [3]. Moreover, previous cross-sectional clinical investigations on this topic have not been able to directly confirm that tirzepatide produces significant amounts of food residues.

Although this is a single case report, it is still a valuable and informative case of an esophagogastroduodenoscopy performed before the use of tirzepatide, 2 esophagogastroduodenoscopies conducted under the circumstances of using tirzepatide, and an esophagogastroduodenoscopy performed after tirzepatide was discontinued for 2 months. The findings are significant because they show unequivocally that tirzepatide leaves food residues that make esophagogastroduodenoscopy challenging to perform when used as part of a standard food regimen, which involves fasting for at least 12 hours before the procedure and only permitting water consumption. Furthermore, this study also confirms that a large amount of food residue is a reversible phenomenon that disappears with the discontinuation of tirzepatide.

The findings of this study suggest the need for further research on the optimal duration of tirzepatide cessation prior to esophagogastroduodenoscopy or procedures requiring anesthesia and balancing this with the risk of loss of glycemic control.

## Learning Points

- Tirzepatide is a drug widely used to treat T2D, obesity, and sleep apnea syndrome due to severe obesity.
- Care should be taken when performing esophagogastroduodenoscopy on a patient using tirzepatide because normal pretreatment may result in residual contents in the stomach.
- It is suggested that tirzepatide be deactivated beforehand when performing esophagogastroduodenoscopy on patients using tirzepatide.

## Data Availability

Original data generated and analyzed for this case report were included in this published article.
